# Paravertebral Triangle of Dulness in Pleural Effusion

**Published:** 1907-04

**Authors:** 


### PARAVERTEBRAL TRIANGLE OF DULNESS IN PLEURAL EFFUSION.

Thayer and Fabyan (Am. J. Med. Sci., January, 1907) call attention to this valuable sign described by Grocco in 1902.

It is a triangular dulness on the side opposite the effusion. The apex is about the height of the fluid at the vertebarl column the base extends two to seven c.m. from the spine. The vertical side of the triangle is represented by the line of the apophyses of the vertebrae. The hypothenuse connects the two and sometimes shows a slight convexity.

When the patient lies on the affected side the triangle of dulness diminishes or disappears, and reappears again when the erect posture is assumed, or when the patient lies on the other side.

The triangle is usually larger on the left side—that is in the case of right sided effusion—than on the right.

This conclusion, too, is reached, that although there may be a small strip of dulness along the spine on the side opposite a pneumonia, they have but once detected anything approaching a paravertebral triangle—this showed little or no change of position.

ions, displacements, subinvolution, fibroids, and other neoplasms is caused by the accompanying chronic endometritis, which prevents proper implantation. (6) A cause of unhealthy endometrium and of tubal disease which may prevent union of the male and female elements in the chronic inflammation and congestion of the uterus and adnexa incident to subinvolution and sepsis. (7) Gonorrhea not only causes sterility in women in seventy per cent. of cases, but is also the cause of sterility in the male. (8) In two-thirds of all cases of gonorrhea in women, the woman has been infected by the man.

